# Supplementary material for: Immune molecular profiling of a multiresistant primary prostate cancer with a neuroendocrine-like phenotype: a case report
Source: BMC Urol. 2020 Oct 28;20:171. doi: 10.1186/s12894-020-00738-8 (PMC7592533; doi:10.1186/s12894-020-00738-8)
Supplement: Supplementary file 1 — Additional file 1. Supplementary methods for tissue collection, histology and staining, immune profiling, molecular characterization, genomic profiling, and bioinformatic approaches. [file 12894_2020_738_MOESM1_ESM.docx]

**Supplementary Methods**

*Tissue collection and processing*

Tissue collection methods were varied amongst the four samples and comprised of either transrectal core biopsy (single biopsy 18G; *diagnostic*), transperineal core biopsies (five 16G cores; *RT-* and four 16G cores; *RT+*). The recurrence biopsy was a selected block of FFPE tissue from transurethral resection (TURP; *Recurrence*). Fresh tissue from each patient sample was fixed immediately by immersion in a solution of 10% neutral buffered formaldehyde prior to paraffin-embedding.

*Histology and staining*

3 µm sections were collected and used for histopathological analysis including H&E and DAB stains. Staining for neuroendocrine histology used antibodies for synaptophysin (SYN: NCL-L-SYNAP-299, Leica Novocastra), neuron-specific enolase (NSE: Clone BBS/NC/VI-H14, Dako), thyroid transcription factor-1 (TTF-1: Clone SP141, Ventana), chromogranin (CG; Clone DAK-A3, Dako), CD56 (MRQ-42, Cell Marque) and androgen receptor (AR: Clone AR441, Dako). Dual colour IHC was performed on a Ventana Benchmark ULTRA autostainer using CD3 (Clone SP7, Abcam) with OptiView DAB and CD45 (Clone 2B11+DP7/26, Dako) with ultraView Universal AP detection systems

*Immune profiling and cell counting*

High resolution scans of dual-color CD3/CD45 stains were uploaded into Halo (Indica Labs) software for densitometry analysis. Whole tissue sections for all four samples were spatially segmented within the software according to histopathological classification of tumor-bearing, tumor-stroma, and mixed tissue zones by a trained pathologist (Figure 2A). Cells were counted within either the tumor stroma or tumor zones according to two cell phenotypes: (i) CD45^+^ CD3^-^, and (ii) CD3^+^, representing non-lymphocytic leucocytes and lymphocytic T cells, respectively. The number of identified cells in each tissue zone was then normalized to the total surface area (Table 1) to account for differences in tissue size.

*Transcriptomics and proteomics analyses*

10 µm sections of formalin-fixed tissue blocks were obtained for downstream RNA and protein extracted for transcriptomic and proteomics analysis, respectively, as per previous studies [1, 2]. This generated normalized mRNA and protein expression levels across the four samples. As input for GSEA analysis of tissue comparisons, normalised data was ranked by magnitude of difference between pairs of samples. For recurrence tissue, all comparisons were made directly to diagnostic tissue (i.e. Recurrence – Diagnostic) in absence of androgen deprivation and radiation therapy.

*Genomic CNA profiling*

Total genomic DNA was extracted from 10µm sections of formalin-fixed tissue blocks using QIAamp DNA FFPE tissue kit (Qiagen; Cat: 56404). The total amount of DNA obtained for each biopsy section was 20.7ng (diagnostic), 55ng (RT-), 51.5ng (RT+) and 112ng (recurrence). DNA libraries were prepped for sequencing using NEBNext Ultra II (NEB) kit and sequenced using a NextSeq HO 75PE (Illumina) run. The number of sequences used as input for CNA analysis was 10,815,543 (diagnostic). 6,023,339 (RT-), 8,166,806 (RT+), and 3,983,279 (recurrence).Raw Bam files were analysed for CNA variation using the unaltered R package *QDNAseq* [3]. Parameters for analysis were a binSize of 500 with CNA calls made with “cutoff” method. Called bins were subsequently exported in .igv format for heatmap generation and gene location identification in IGV using genome version hg19.

*Bioinformatic analyses*

Fold changes in transcripts/proteins were calculated relative to diagnostic level. Heatmaps were generated using Perseus computational platform using z-score row-normalised log- expression values. Gene set enrichment analysis (GSEA) was performed using Broad GSEA software, and Molecular Signature Database (MSigDB) according to default parameters and established protocols [4]. An FDR of <0.2 was considered significant for GSEA pathway analysis. Output data was visualised using custom *ggplot* R script.

1. Keam, S.P., et al., *The Transcriptional Landscape of Radiation-Treated Human Prostate Cancer: Analysis of a Prospective Tissue Cohort.* Int J Radiat Oncol Biol Phys, 2018. **100**(1): p. 188-198.

2. Keam, S.P., et al., *Exploring the oncoproteomic response of human prostate cancer to therapeutic radiation using data-independent acquisition (DIA) mass spectrometry.* Prostate, 2018. **78**(8): p. 563-575.

3. Scheinin, I., et al., *DNA copy number analysis of fresh and formalin-fixed specimens by shallow whole-genome sequencing with identification and exclusion of problematic regions in the genome assembly.* Genome Res, 2014. **24**(12): p. 2022-32.

4. Subramanian, A., et al., *Gene set enrichment analysis: a knowledge-based approach for interpreting genome-wide expression profiles.* Proc Natl Acad Sci U S A, 2005. **102**(43): p. 15545-50.
